# The association between YouTube use and knowledge of human papillomavirus-related cancers

**DOI:** 10.1016/j.pecinn.2023.100186

**Published:** 2023-06-30

**Authors:** Vanessa G. Dorismond, W. John Boscardin, George F. Sawaya

**Affiliations:** a Department of Obstetrics and Gynecology, University of California, San Francisco, CA, USA; b Department Epidemiology & Biostatistics, University of California, San Francisco School of Medicine, 490 Illinois St, San Francisco, CA 94158, USA; c Department of Obstetrics and Gynecology, University of California, San Francisco School of Medicine, 2356 Sutter St, San Francisco, CA 94115, USA

**Keywords:** Human papillomavirus^e^, HPV, HPV-related cancers, Cervical cancer, HPV-related cancer knowledge, Knowledge

## Abstract

**Objective:**

To examine the association between YouTube usage and HPV-related cancer knowledge (cervical, anal, oral and penile).

**Study design:**

Cross-sectional study using data from the Health Information National Trends survey conducted between 2017 and 2020 (*N* = 16,092). Logistic regression was used to analyze the independent effect of YouTube use on cancer knowledge, controlling for sociodemographic characteristics.

**Results:**

Respondents' knowledge of HPV-related cancers varied: 49.9% about cervical, 18% anal, 20.1% oral and 20.4% penile cancers. YouTube use was associated with increased knowledge for all cancers (cervical: OR 2.66, 95% CI 2.04, 3.46; anal: OR 1.83, 95% CI 1.32, 2.53; oral: OR 1.89, 95% CI 1.37, 2.61; penile OR 2.00, 95% CI 1.44, 2.77) in models adjusted for all covariates. Other independent predictors of HPV-related cancer knowledge included female gender, younger age, a higher income, and higher education.

**Conclusion(s):**

YouTube could play an important role in educating people about HPV-related cancers and should also target other populations, such as males and those with less formal education.

**Innovation:**

The study provides novel insights into the potential of YouTube as an educational tool for promoting cancer knowledge with the goal of cancer prevention.

## Highlights

• Novel investigation of YouTube use as an important resource for patient education.
• Cross sectional study utilizing large sample data set from HINTS 5 (2017–2020).
• YouTube use is an independent predictor of HPV-related cancer knowledge.
• Gender, income, education as independent predictors of HPV-related cancer knowledge.
• Targeting other high-risk groups and non-Internet-based platforms remains crucial.

## 1 Introduction

In 2018, the prevalence of human papillomavirus (HPV) infections in the United States was estimated to be 43 million, with 13 million being new infections [1,2]. Although most HPV subtypes are cleared by the body without consequence, oncogenic HPV subtypes cause most cervical, anal, oropharyngeal, vaginal, vulvar, and penile cancers [[1], [2], [3], [4], [5], [6], [7], [8]].  Widespread HPV vaccination has the potential to reduce cervical cancer incidence worldwide by as much as 90% while also reducing the need for screening and subsequent medical care, biopsies, and invasive procedures [5].  Vaccination also has the potential to protect against penile, anal and oropharyngeal cancers [1] .

The Healthy People 2020 goal to have at least 80% of adolescents aged 13–15 years receive the Advisory Committee on Immunization Practices recommended number of appropriately spaced doses of HPV vaccine fell short, with only a rate of 54.5% by 2020 [9]. One possible approach to address this concern is through increasing awareness and knowledge about HPV infection and vaccination. Several research studies have demonstrated that adequate understanding regarding HPV can lead to an increase in interest, acceptance, intention to get vaccinated, as well as actual uptake rates for the HPV vaccine [[10], [11], [12], [13], [14], [15], [16]]. With social medial playing a significant role among those who access health information online, it presents an avenue where programs could reach out specifically aiming towards educating more people about the need for receiving the HPV vaccine. In fact, a survey conducted by Pew Research Center in 2021, found that YouTube is the most commonly used social media platform (81% of US adults, effectively 95% of those 18–29 years old use the platform) [17].  In addition, a study using Health Information National Trends Survey (HINTS) data from 2020 found that 40.8% of US adult respondents use YouTube to watch health-related videos [18]. A systematic review showed that many individuals are willing and interested in receiving accurate and helpful information regarding HPV and HPV vaccination via social media and are more comfortable doing so online than in-person [19]. As such, YouTube and the Internet impact the awareness, knowledge and attitudes in which individuals consume information, including HPV and HPV vaccination information [19].

Our objective was to examine the association between Internet and YouTube usage on knowledge of HPV-related cancers and to explore the role of underlying sociodemographic characteristics on this association. Understanding these characteristics will allow for the creation of interventions geared towards specific sociodemographic groups to improve HPV vaccine uptake.

## 2 Material and methods

### 2.1 Data source

We used data obtained from HINTS 5 (Cycles 1–4, total sampled *N* = 66,723), a cross-sectional study of civilian, non-institutionalized adults 18 years or older living in the United States. HINTS examines trends in health information usage over time and provides data for conducting fundamental research on associations among cancer-related communication, knowledge, attitudes, and behavior at the population level. Data were obtained from HINTS and prepared for the National Cancer Institute by Westat. Cycle 1 data (total respondents *n* = 3285) were collected from January 2017 until May 2017; Cycle 2 data (*n* = 3504), from January 2018 until May 2018; Cycle 3 data (*n* = 5438) from January 2019 until May 2019; and Cycle 4 data (*n* = 3865) from January 2020 until June 2020. All households received a mailing of the survey either in English or in Spanish, and one adult in the household completed the survey. Details about the HINTS data source and methodology are available on the HINTS website [20].

### 2.2 Measures

The primary predictor was a trichotomous variable created as a combination of the Internet use and YouTube use variables. Internet use status was determined by answering the following question: Do you ever go on-line to access the Internet or world wide web. Or to send and receive email? A “yes” answer equated to an Internet user, a “no” answer equated to a non-Internet user. The YouTube use status was determined by the following question: In the past 12 months, have you used the Internet for any of the following reasons? Watched a health-related video on YouTube? A “yes” answer equated to a YouTube user, a “no” answer equated to what we subsequently referred to as a non-YouTube user. By combining these two variables, we created a trichotomous variable with the following subgroups: 1) non-Internet users/non-YouTube users; 2) Internet users/non-YouTube users; 3) Internet users/YouTube users. For simplicity, we use the term “YouTube user” to describe this last group henceforth.

Sociodemographic characteristics (covariates) assessed were gender (male/female); race and ethnicity (non-Hispanic White [NHW], non-Hispanic Black [NHB], Hispanic, or Other); age, marital status, education level, income level, insurance, whether they had regular health provider, general health, and number of children under 18 in the household. Table 1 details additional categorization of these covariates.

**Table 1:** Sociodemographic characteristics of respondents to HINTS^a^ 5 Cycles 1–4 (2017–2020).

Characteristics	Sample^a^, n (%) Total *N* = 16,092	Weighted^b^ sample, n	Weighted proportion, %
Age, years	15,585	244,090,733	
65+	5671 (36.4)	48,495,969	19.9
50–64	4986 (32.0)	72,779,852	29.8
35–49	2984 (19.2)	64,227,318	25.3
18–34	1944 (12.5)	58,587,595	24.0
Race and Ethnicity	14,444	230,773,671	
Non-Hispanic Black	2011 (13.9)	25,137,435	10.9
Non-Hispanic White	9038 (62.6)	148,410,333	64.3
Hispanic	2214 (15.3)	37,821,463	16.4
Other	1181 (8.2)	19,404,441	8.4
Gender	15,739	246,102,652	
Male	6494 (41.3)	120,049,285	48.8
Female	9245 (58.74)	126,053,368	51.2
Marital status	15,603	244,523,907	
Never married	2644 (17.0)	74,409,735	30.4
Divorced/Widowed/Separated	4636 (29.7)	36,734,386	15.0
Married/Living as Married	8323 (53.3)	133,378,786	54.6
Education	15,637	245,182,516	
Less than high school	1099 (7.0)	20,017,939	8.2
High school graduate	2898 (18.5)	55,881,946	22.8
Some college / post high school training	4653 (29.8)	93,273,853	38.0
College graduate/postgraduate	6987 (44.7)	76,008,877	31.0
Income	14,295	227,104,295	
$0 to $19,999	2666 (18.7)	38,950,724	17.2
$20,000 to $34,999	1916 (13.4)	26,433,621	11.6
$35,000 to $49,999	1880 (13.2)	30,979,910	13.6
$50,000 to $74,999	2537 (17.8)	41,226,420	18.2
$75,000 or more	5296 (37.1)	89,513,620	39.4
Regular provider	15,823	246,972,651	
No	4,683,929.6)	87,939,376	35.6
Yes	11,140 (70.4)	159,033,275	64.4
Health Insurance	15,835	247,079,586	
No	832 (5.25)	21,132,357	8.6
Yes	15,003 (94.8)	225,947,230	91.4
General health	15,871	248,364,521	
Fair/Poor	2619 (16.5)	37,895,446	15.3
Good	5674 (35.8)	87,447,656	35.2
Very good/Excellent	7578 (47.8)	123,021,417	49.5
Number of children under 18 in household	14,810	234,632,194	
0	11,211 (75.7)	159,540,724	68.0
1+	3599 (24.3)	75,091,470	32.0
Internet/YouTube use	15,364	240,990,110	
Non-Internet users/Non-YouTube users	2773 (18.1)	33,870,526	14.1
Internet users/Non-YouTube users	7849 (51.1)	123,763,699	51.4
YouTube users	4742 (30.9)	83,355,885	34.6

*HINTS:* Health Information Trends Survey.

a Total number N of complete responses for each variable reflects non-missing data.

b Population level estimates.

### 2.3 Outcome

**Fig. 1:**
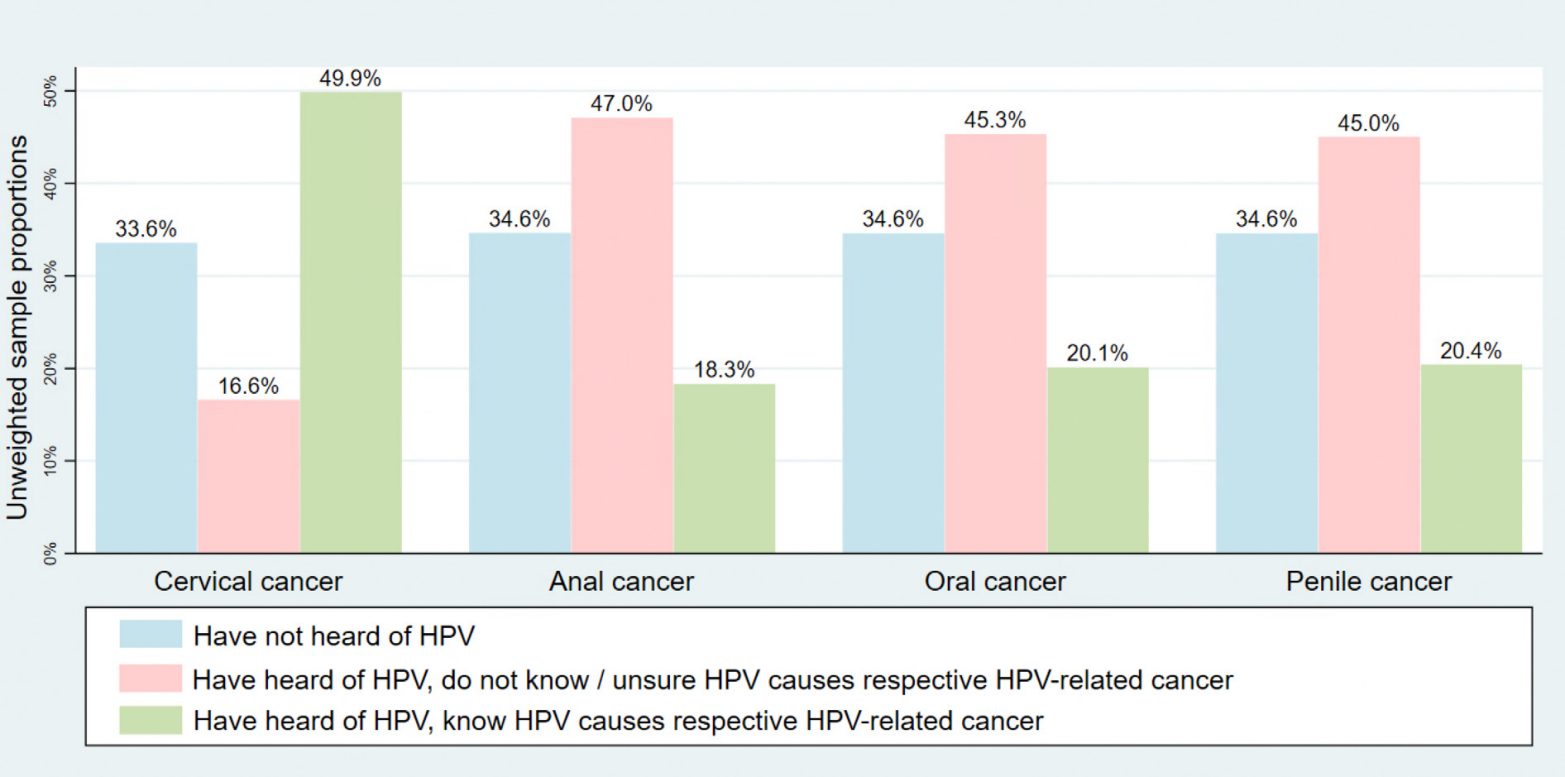
Knowledge of HPV-related cancers by cancer group in the population. This figure depicts the unweighted distribution of respondents for each HPV-related cancer question. “Have not heard of HPV” (blue) was considered a skip question in the overall survey. As such, for those who have heard of HPV, each respondent was then asked whether they know that HPV causes the respective cancer. These results are also depicted in row 1 of Table 2. (For interpretation of the references to color in this figure legend, the reader is referred to the web version of this article.) *HPV*, human papillomavirus

**Fig. 2:**
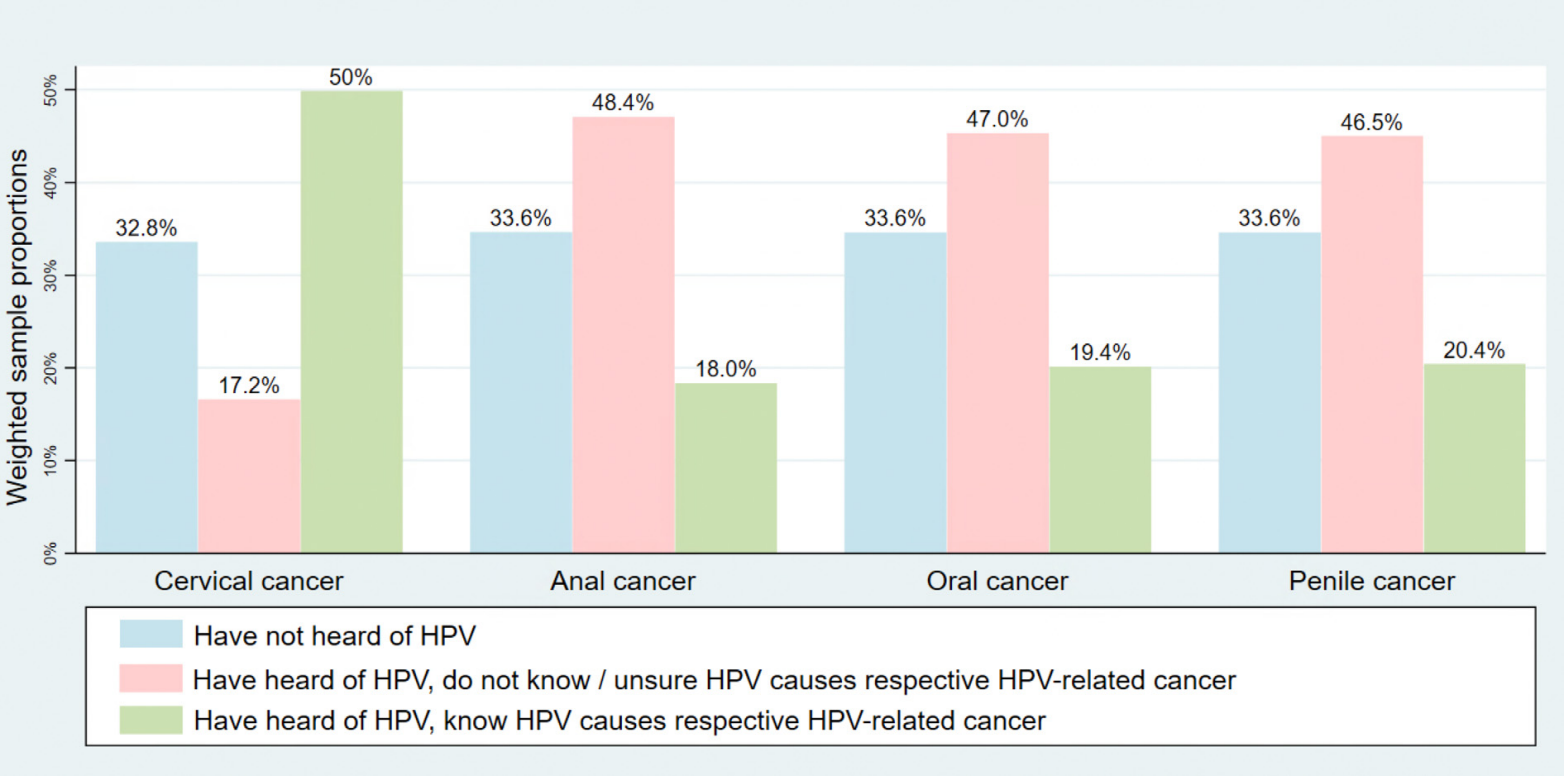
Knowledge of HPV-related cancers by cancer group in the weighted population. This figure depicts the weighted distribution of respondents for each HPV-related cancer question. “Have not heard of HPV” (blue) was considered a skip question in the overall survey. As such, for those who have heard of HPV, each respondent was then asked whether they know HPV causes the respective cancer. These results are also depicted in row 1 of Table 2. (For interpretation of the references to color in this figure legend, the reader is referred to the web version of this article.) *HPV*, human papillomavirus

The primary outcome was knowledge of HPV-related cancers, assessed by a “yes” response to each of the following questions: 1) Do you think that HPV can cause cervical cancer? 2) Do you think that HPV can cause anal cancer? 3) Do you think that HPV can cause oral cancer? 4) Do you think that HPV can cause penile cancer? Answering either “no” or “not sure” on any question equated to not being knowledgeable about the respective HPV-related cancer; answering “yes” equated to being knowledgeable to HPV-related cancers. We restricted our analysis to respondents who answered “yes” to the preceding question “Have you heard of HPV?”; respondents who answered “no” were not asked any of the four HPV-related questions and were categorized as “Have not heard of HPV” (Table 2, Table 3, Fig. 1, Fig. 2).

**Table 2:** Sociodemographic characteristics of those who are knowledgeable regarding HPV-related cancers.

Characteristics	Cervical cancer			Anal cancer			Oral cancer			Penile cancer		
	Sample^a^, n(%)	*p*^b^-value	Weighted^c^ sample, n(%)	Sample, n(%)	*p-*value	Weighted sample, n(%)	Sample, n(%)	*p-*value	Weighted sample, n(%)	Sample, n(%)	*p-*value	Weighted sample, n(%)
Percent of total respondents who answered “Yes” to the respective HPV-related cancer question	49.9%		50.0%	18.3%		18.0%	20.1%		19.4%	20.4		19.9%
Age, years		<0.001			<0.001			<0.001			<0.001	
65+	1788 (34.3)		141.63 (31.8)	682 (13.5)		54.19 (12.4)	763 (15.1)		61.31 (14.1)	783 (15.5)		63.29 (14.5)
50–64	2372 (50.9)		321.45 (47.2)	898 (19.8)		118.91 (18.0)	978 (21.5)		127.17 (19.2)	974 (21.4)		128.05 (19.3)
35–49	1894 (66.0)		370.57 (60.2)	644 (22.8)		128.68 (21.3)	714 (25.3)		136.71 (22.6)	720 (25.5)		142.66 (23.6)
18–34	1285 (68.2)		327.22 (57.8)	416 (22.2)		110.04 (19.5)	450 (24.0)		118.59 (21.1)	457 (24.4)		120.53 (21.4)
Race and Ethnicity		<0.001			<0.001			<0.001			<0.001	
Non-Hispanic Black	795 (44.0)		101.04 (44.3)	286 (16.1)		32.11 (14.4)	281 (15.9)		33.18 (15.0)	352 (19.9)		43.89 (19.7)
Non-Hispanic White	4838 (55.6)		790.28 (55.1)	1725 (20.2)		280.03 (19.8)	1945 (22.8)		306.24 (21.7)	1878 (22.0)		302.20 (21.4)
Hispanic	938 (45.5)		162.37 (46.0)	334 (16.7)		58.09 (16.9)	355 (17.7)		59.15 (17.2)	379 (18.9)		63.63 (18.5)
Other	526 (46.1)		78.12 (41.7)	203 (18.2)		30.98 (16.8)	218 (19.6)		33.06 (18.0)	226 (20.3)		31.16 (16.9)
Gender		<0.001			<0.001			<0.001			<0.001	
Male	2352 (38.5)		452.42 (39.8)	927 (15.3)		173.97 (15.5)	1038 (17.2)		185.26 (16.6)	1013 (16.8)		185.25 (16.5)
Female	5053 (58.1)		715.95 (59.9)	1738 (20.6)		239.38 (20.5)	1894 (22.4)		260.75 (22.3)	1945 (23.0)		270.16 (23.1)
Marital Status		<0.001			<0.001			<0.001			<0.001	
Never married	1329 (53.7)		354.36 (50.3)	475 (19.5)		129.29 (18.6)	507 (20.8)		136.38 (19.6)	519 (21.3)		137.61 (19.8)
Divorced/Widowed/Separated	1774 (41.8)		143.45 (42.4)	676 (16.4)		52.06 (15.9)	724 (17.5)		57.96 (17.6)	746 (18.1)		59.99 (18.3)
Married/Living as Married	4250 (53.5)		664.42 (52.2)	1498 (19.3)		231.42 (18.5)	1527 (21.7)		251.10 (20.1)	1680 (21.6)		258.03 (20.7)
Education		<0.001			<0.001			<0.001			<0.001	
Less than high school	202 (21.8)		39.39 (22.8)	79 (8.7)		15.75 (9.2)	80 (8.8)		14.98 (8.8)	85 (9.4)		18.44 (10.8)
High school graduate	792 (30.5)		161.29 (31.4)	288 (11.4)		57.30 (11.5)	299 (11.8)		58.9 (11.8)	312 (12.3)		61.94 (12.4)
Some college/Post high school training	2049 (46.4)		467.60 (52.2)	726 (16.9)		164.08 (18.7)	795 (18.5)		173.87 (19.8)	870 (20.2)		191.05 (21.7)
College graduate/Postgraduate	4331 (64.1)		497.47 (67.3)	1564 (23.6)		176.58 (24.3)	1750 (26.4)		197.72 (27.2)	1685 (25.4)		184.98 (25.4)
Income		<0.001			<0.001			<0.001			<0.001	
$0 to $19,999	805 (34.3)		126.04 (35.9)	288 (12.6)		48.57 (14.1)	299 (13.1)		50.91 (14.7)	342 (15.0)		56.41 (16.4)
$20,000 to $34,999	716 (40.4)		100.85 (41.3)	264 (15.3)		38.20 (15.9)	278 (16.1)		40.68 (17.0)	285 (16.5)		41.81 (17.5)
$35,000 to $49,999	808 (45.9)		139.01 (47.9)	299 (17.3)		53.13 (18.6)	322 (18.6)		51.67 (18.2)	330 (19.1)		53.81 (18.9)
$50,000 to $74,999	1280 (52.3)		191.79 (48.3)	456 (19.1)		63.89 (16.3)	490 (20.5)		66.98 (17.1)	507 (21.2)		70.96 (18.1)
$75,000 or more	3318 (64.3)		542.22 (62.3)	1194 (23.6)		189.19 (22.1)	1360 (26.8)		210.82 (24.6)	1321 (26.0)		210.53 (24.5)
Regular Provider		<0.001			<0.001			<0.001			<0.001	
No	1956 (46.0)		368.74 (45.2)	686 (16.4)		127.72 (15.9)	745 (17.8)		133.00 (16.6)	777 (18.6)		143.57 (17.9)
Yes	5432 (51.7)		797.46 (52.9)	1978 (19.3)		285.72 (19.3)	2180 (21.2)		312.06 (21.1)	2180 (21.3)		311.24 (21.1)
Health Insurance		<0.001			<0.001			<0.001			<0.001	
No	319 (43.0)		84.81 (44.1)	99 (13.5)		29.36 (15.6)	106 (14.5)		27.26 (14.5)	119 (16.3)		30.77 (16.4)
Yes	7065 (50.4)		108.11 (50.7)	2555 (18.6)		383.91 (18.3)	2812 (20.5)		417.80 (20.0)	2829 (20.6)		424.78 (20.3)
General Health		<0.001			<0.001			<0.001			<0.001	
Fair/Poor	868 (37.2)		134.28 (38.9)	332 (14.5)		52.10 (15.3)	344 (15.0)		55.12 (16.2)	386 (16.9)		61.25 (18.0)
Good	2425 (45.7)		385.86 (47.0)	865 (16.7)		135.09 (16.9)	926 (17.9)		137.63 (22.0)	963 (18.6)		144.62 (18.1)
Very good/Excellent	4109 (57.2)		649.39 (55.4)	1466 (20.8)		227.31 (19.7)	1657 (23.6)		253.83 (22.0)	1604 (22.8)		250.55 (21.7)
Number of children under 18 in household		<0.001			<0.001			<0.001			<0.001	
0	5054 (47.9)		727.55 (48.0)	1823 (17.7)		249.19 (16.8)	2034 (19.7)		275.18 (18.5)	2004 (19.4)		272.13 (18.3)
1+	2123 (61.6)		409.17 (57.3)	747 (22.0)		153.01 (21.7)	797 (23.5)		157.15 (22.3)	863 (25.5)		171.90 (24.4)
YouTube / Internet users		<0.001			<0.001			<0.001			<0.001	
Non-Internet users, Non-YouTube users	453 (19.2)		52.34 (17.9)	186 (8.1)		23.14 (8.0)	186 (8.1)		23.44 (8.1)	195 (8.5)		24.53 (8.5)
Internet users, Non-YouTube users	3936 (52.8)		602.95 (51.3)	1350 (18.5)		204.21 (17.7)	1524 (20.9)		224.53 (19.5)	1524 (20.9)		225.25 (19.5)
YouTube users	2875 (63.1)		491.95 (61.1)	1086 (24.4)		176.72 (22.4)	1176 (26.3)		188.68 (23.9)	1188 (26.6)		194.31 (24.6)

*HPV,* human papillomavirus.

a Percentage of yes out of a total of yes, no/not sure, and “Have not heard of HPV” data.

b *p*-value for chi-square test for each covariate and its association to each HPV-cancer related question (all answer choices included).

c Population estimate (n) x 100,000.

**Table 3:** Logistic regression model of independent predictors for HPV-related cancer knowledge among knowledgeable participants.

Characteristics	Cervical cancer		Anal cancer		Oral cancer		Penile cancer	
	Adjusted OR^a^	95% CI	Adjusted OR^a^	95% CI	Adjusted OR^a^	95% CI	Adjusted OR^a^	95% CI
Age, years								
65+	*Ref*		*Ref*		*Ref*		*Ref*	
50–64	1.63	1.41, 1.89	1.26	1.03, 1.56	1.19	0.99, 1.44	1.09	0.91, 1.29
35–49	2.80	2.29, 3.40	1.24	0.95, 1.61	1.23	0.97, 1.56	1.14	0.90, 1.46
18–34	2.57	2.05, 3.22	1.11	0.81, 1.53	1.10	0.84, 1.43	1.08	0.87, 1.34
Race and Ethnicity								
Non-Hispanic Black	*Ref*		*Ref*		*Ref*		*Ref*	
Non-Hispanic White	1.43	1.11, 1.83	1.49	1.16, 1.92	1.40	1.07, 1.84	1.13	0.91, 1.41
Hispanic	1.07	0.82, 1.41	1.32	0.97, 1.82	1.26	0.89, 1.77	1.04	0.81, 1.35
Other	0.62	0.44, 0.87	1.14	0.79, 1.65	1.08	0.75, 1.55	0.79	0.57, 1.11
Gender								
Male	*Ref*		*Ref*		*Ref*		*Ref*	
Female	2.74	2.37, 3.16	1.35	1.14, 1.61	1.41	1.21, 1.64	1.47	1.26, 1.72
Marital status								
Never married	*Ref*		*Ref*		*Ref*		*Ref*	
Divorced/Widowed/Separated	1.13	0.86, 1.47	0.94	0.68, 1.29	0.99	0.72, 1.36	1.07	0.83, 1.39
Married/Living as Married	1.03	0.83, 1.29	0.81	0.60, 1.09	0.85	0.64, 1.23	0.94	0.74, 1.19
Education								
Less than high school	*Ref*		*Ref*		*Ref*		*Ref*	
High school graduate	1.40	0.95, 2.07	1.26	0.74, 2.12	1.24	0.75, 2.06	1.15	0.71, 1.88
Some college/Post high school training	2.76	1.95, 3.93	2.00	1.27, 3.13	1.90	1.21, 2.99	2.02	1.27, 3.20
College graduate/Postgraduate	4.33	2.97, 6.31	2.54	1.59, 4.05	2.56	1.60, 4.12	2.18	1.37, 3.46
Income								
$0 to $19,999	*Ref*		*Ref*		*Ref*		*Ref*	
$20,000 to $34,999	1.04	0.78, 1.39	1.12	0.81, 1.56	1.07	0.77, 1.50	1.02	0.76, 1.38
$35,000 to $49,999	1.31	0.96, 1.79	1.23	1.23	1.11	0.80, 1.55	1.01	0.71, 1.44
$50,000 to $74,999	1.20	0.89, 1.62	1.07	0.79, 1.45	0.99	0.72, 1.37	0.99	0.73, 1.34
$75,000 or more	1.63	1.26, 2.09	1.27	0.91, 1.79	1.24	0.90, 1.72	1.24	0.93, 1.65
Regular Provider								
No	*Ref*		*Ref*		*Ref*		*Ref*	
Yes	1.43	1.22, 1.68	1.25	1.04, 1.51	1.34	1.12, 1.61	1.20	1.00, 1.43
Health Insurance								
No	*Ref*		*Ref*		*Ref*		*Ref*	
Yes	0.89	0.60, 1.31	0.84	0.53, 1.34	0.99	0.65, 1.50	0.97	0.69, 1.37
General Health								
Fair/Poor	*Ref*		*Ref*		*Ref*		*Ref*	
Good	1.01	0.80, 1.27	0.96	0.71, 1.30	0.88	0.68, 1.15	0.80	0.64, 1.01
Very good/Excellent	1.10	0.85, 1.43	0.96	0.74, 1.25	1.00	0.78, 1.28	0.90	0.72, 1.11
Number of children under 18 in household								
0	*Ref*		*Ref*		*Ref*		*Ref*	
1+	1.09	0.92, 1.31	1.39	1.17, 1.65	1.23	1.00, 1.51	1.39	1.16, 1.67
YouTube / Internet users								
Non-Internet users, Non-YouTube users	*Ref*		*Ref*		*Ref*		*Ref*	
Internet users, Non-YouTube users	2.02	1.56, 2.60	1.43	1.07, 1.92	1.59	1.16, 2.17	1.57	1.16, 2.11
Internet users, YouTube users	2.66	2.04, 3.46	1.83	1.32, 2.53	1.89	1.37, 2.61	2.00	1.44, 2.77

*HPV,* human papillomavirus; *OR,* Odds ratio; *CI*: Confidence interval.

a OR adjusted for all variables in the table; includes 1. “have heard of HPV”, 2. have heard of HPV / “yes, know HPV causes respective cancer”, 3. have heard of HPV / “no/not sure HPV causes respective cancer” data.

### 2.4 Statistical analysis

All analyses were performed using STATA version 17.1. Unweighted and population-weighted estimates (n, %) were calculated for all participant characteristics and outcome prevalence. A weighted multivariate logistic regression model was performed to assess the independent association of HPV knowledge among YouTube users compared with non-Internet users/non-YouTube users and to assess whether sociodemographic characteristics independently predicted HPV knowledge regarding HPV-related cancers. We included all aforementioned covariates in this analysis. All weighted analyses accounted for the complex survey design using jackknife replication as documented in the HINTS technical manuals. Statistical significance was a two-sided *p* < 0.05 with no formal adjustment for multiple comparisons.

## 3 Results

Table 1 shows the demographic characteristics of the sample and the weighted population estimates. The average age of sample respondents was 54.8 years. Approximately 60% of respondents were female and 63% identified as NHW; 18% were non-Internet/non-YouTube users and about 31% used YouTube. A similar trend was observed across the weighted population estimates.

Fig. 1, Fig. 2 show the knowledge of HPV-related cancers by cancer type in the unweighted and weighted sample populations respectively. As seen in Fig. 1 (and similarly for Fig. 2), half of respondents (49.9%) knew that HPV causes cervical cancer, but fewer were knowledgeable about whether the other three cancers were HPV-related (18.3% for anal, 20.1% for oral, and 20.4% for penile). Approximately one third of all respondents had not heard of HPV.

Table 2 shows sociodemographic characteristic differentials among those knowledgeable regarding HPV-related cancers. For all cancers, knowledge was highest among YouTube users, as well as those of younger age, NHW race and ethnicity, female gender, higher education, a combined household income $75,000 or more, having a regular provider, and having more than one child under age 18 in the household. Comparatively, across all sociodemographic characteristics, most respondents were not knowledgeable regarding HPV-related anal, oral, or penile cancers.

Table 3 shows results of multivariate analyses identifying independent predictors of knowledge about HPV-related cancers adjusted for all other covariates. For cervical cancer, being a YouTube user was an independent predictor of knowledge after controlling for potential confounders (odds ratio [OR] 2.66, 95% confidence interval [CI] 2.04–3.46). Compared to NHB participants, NHW had higher odds of knowing that HPV causes cervical cancer (OR 1.43, 95% CI 1.11–1.83) and “Other” race and ethnicity had lower odds (OR 0.62, 95% CI 0.44–0.87).

Independent predictors for HPV-related anal and oral cancer knowledge were similar with a few exceptions. For anal cancer, those with one or more children under age 18 in the household (OR 1.39, 95% CI 1.17–1.65) had increased knowledge compared to those without children, but no effect was seen with race and ethnicity. For oral cancer, both an effect of having children in the household (OR 1.23; 95% CI 1.00–1.51) and NHW race and ethnicity (OR 1.40, 95% CI 1.07–1.84) were noted; no knowledge association was apparent for age. For HPV-related penile cancer, no significant associations were noted between knowledge and age, race/ethnicity, income or having a regular provider. However, having one or more child in the household was associated with increased odds of knowledge (1.39, 95% CI 1.16–1.67). Similar to HPV-related cervical cancer, YouTube use was associated with the highest increased odds of knowledge regarding HPV-related cancers (anal cancer OR 1.83, 95% CI 1.32–2.53; oral cancer OR 1.89, 95% CI 1.37–2.61; penile cancer OR 2.00, 95% CI 1.44–2.77).

## 4 Discussion and conclusion

### 4.1 Discussion

#### 4.1.1 Principal findings

We found that most respondents who have heard of HPV knew that HPV causes cervical cancer but relatively few knew of its relationship to anal, oral, and penile cancers. Though our analysis showed statistically significant knowledge gaps across various sociodemographic characteristics, this overall knowledge is consistent with previously published studies [21].  Our study uniquely highlights YouTube usage as an independent predictor of HPV-related cancer knowledge among respondents who know that HPV causes the respective cancers. By doing so, we associate YouTube as an important health information resource contributing to the knowledge regarding HPV-related cancers. This research is also important for exploring the characteristics of those with the most knowledge regarding HPV-related cancers and their association to health information both on and off the Internet.

#### 4.1.2 Results in the context of what is known

The emphasis on YouTube to distinguish levels of knowledge regarding HPV-related cancers stems from previous studies showing that YouTube is the most used social media platform and approximately 41% of the US adult population watch health-related videos on YouTube [[17], [18], [19]].  A systematic review by Ortiz et al. analyzing the quality of data on various social media platforms regarding HPV found that most videos about the HPV vaccine viewed in 2014 were anti-vaccine videos [19].    Consequently, this online information can be subject to misinformation and/or anti-vaccine rhetoric that could negatively influence vaccine uptake [19]. Nevertheless, the impact of social media on indivdiuals' awareness, knowledge, attitudes and behaviors related to HPV and HPV vaccination is both positive and negative. Though other studies have used HINTS data to explore the use of the Internet and/or social media with its association to HPV awareness and knowledge [[22], [23], [24], [25], [26]], little information is available exploring the association of all four HPV-related cancers with YouTube use while controlling for various sociodemographic characteristics among knowledgeable respondents. The addition of YouTube use shows the highest odds of knowledge across all four HPV-related cancers. Unlike a recent study using 2020 HINTS data that did not find an association between HPV-related cancer knowledge and social media (including YouTube) [22], our analysis showed YouTube use was an independent predictor of HPV-related cancer knowledge. These differences in findings can be attributed to several reasons: 1) we used four years of HINTS data, thereby increasing the sample size and power of our study, 2) we specifically analyzed YouTube usage as a predictor of Internet-based health-information, and 3) we controlled for a greater number of covariates we believed could confound our analysis. Though we cannot conclude increasing YouTube use will lead to increases in HPV vaccine uptake, our study model uniquely depicts the role of YouTube use as an independent health-information resource and its impact on HPV-related cancer knowledge.

Furthermore, we also highlight gender and racial differences in HPV-related cancer knowledge due to the persistent knowledge gap that exists in these subgroups [23,[25], [26]].    Unlike previous studies, we wanted to assess if these sociodemographic characteristics were independent predictors of knowledge of HPV-related cancers when controlling for YouTube usage. Our finding that females were more knowledgeable about HPV-related cancers compared to males was not entirely unexpected given the prevalence of HPV-related cervical cancer and deaths (as of 2018, approximately 311,00 women died from cervical cancer worldwide)^1^ and from other studies [[24], [25]].  This association is further strengthened by the fact that the initial licensing of HPV vaccines targeted cervical cancer prevention [23], and the recommendation to vaccinate males was made 5 years after the recommendation for females [21].  Highlighting the gender gap is critical because >40% of all HPV-related cancers in the US occur in men. Previous studies have shown that men are more willing to accept the vaccine when male-specific HPV-related health outcomes are emphasized [27].  As such, comprehensive information regarding HPV-related cancers is not being widely disseminated and needs to be inclusive of male-predominant cancers as well.

Our study also showed racial and ethnic disparities in knowledge regarding HPV-related cancers, a novel finding that was not shown in a previous study [26],  perhaps due to our more expansive dataset and analyzing our data among YouTube users. Though statistical significance varied across each HPV-related cancer for race and ethnicity, overall, NHW was independently associated with higher odds of knowing that HPV causes cervical cancer, anal cancer and oral cancer compared to NHB.

#### 4.1.3 Limitations and strengths

This study has several limitations. First, we had to create a trichotomous variable that combined Internet use and YouTube use because these questions were not mutually exclusive in the dataset. As such, across all 4 survey years, *n* = 324 respondents (2.1% of the total analyzed sample) identified themselves as non-Internet/YouTube-users. Rather than reclassifying these individuals as Internet users, since, by definition, one must use the Internet to access YouTube, we categorized them as data entered in error and excluded them. Our study was also limited in the depth of knowledge associated with the HPV-related cancers. Specifically, we could only assess whether knowledge was present or absent, but not the quality of that knowledge (whether informative or non-informative). As such, we cannot draw any conclusions regarding whether increased knowledge will lead to increased HPV vaccine uptake. Despite these limitations, our study had significant strengths. Primarily, it is a national, publicly available database designed to represent the United States population which allowed us to extrapolate data at a larger population level. Secondarily, it had a relatively large sample size to explore the association of sociodemographic characteristics with our outcome of interest.

### 4.2 Innovation

#### 4.2.1 Innovation

The novelty in our study lies in our detailed investigation of YouTube use as an important resource for patient education. This methodological approach innovatively utilized the most recently available HINTS data spanning four years, allowing for an increase in power and statistical significance of our analysis not seen in previous studies. By using such a large sample size, not only did we capture a representation of the population that can vary seasonally or cyclically for any number of reasons, but it also helped mitigate any potential confounding variables that can vary from year to year, such as changes in health policies or demographic shifts. In addition, to gain a more comprehensive understanding of the factors that affect knowledge about HPV-related cancers, we focused our attention to those with prior knowledge regarding the cancerous effects of HPV. Using this methodology enabled us to pinpoint particular factors linked with enhanced comprehension of these types of cancers, especially among those with prior knowledge. This information can be valuable in formulating specialized educational strategies aimed at strengthening cancer prevention efforts. In fact, our results show various independent predictors of HPV-related cancer knowledge other than YouTube / Internet use as a reflection of individuals also seeking their health information outside of the Internet. For instance, having a regular health care provider was an important independent predictor of HPV-related cancer knowledge. This finding emphasizes that the onus remains with the healthcare provider to ensure discussing these important health care topics with their patients, especially for those individuals without access to YouTube and/or the Internet. In addition, despite the misinformation that can plague YouTube and the anti-vaccine rhetoric even in the pre-Covid 19 era, there is evidence that knowledge regarding HPV-related cancers is retained and could impact HPV vaccine uptake in the future.

#### 4.2.2 Future direction

Future directions in research include the use of implementation sciences to evaluate which content on platforms such as YouTube is most beneficial in increasing knowledge regarding HPV-related cancers and how that information might best be disseminated. It is a call to researchers to investigate the quality of HPV-related content online, improve the quality of health information disseminated offline since many individuals still seek their health information from other sources (e.g., their community, healthcare provider), and to ensure these messages are culturally appropriate. Additionally, with the introduction of new social media applications, such as Tiktok and Snapchat, it would be innovative for future studies to investigate knowledge and awareness of HPV and HPV vaccine across these platforms as these viewers compromise the predominant HPV-vaccine eligibility age group. Finally, it would be novel to see the association of HPV-related cancer knowledge, YouTube use and HPV vaccine uptake in the time of the Covid-pandemic where the anti-vaccine rhetoric was at an all-time high across social media.

### 4.3 Conclusion

Efforts to educate the public about human papillomavirus-related cancers should include both Internet and non-Internet platforms to target populations such as older individuals, males, and those with low incomes and less formal education. The innovative nature of this research lies in its ability to provide broader insights into the relationship between HPV-related cancers and knowledge while also suggesting that internet-based platforms such as YouTube could play an important role in educating people about HPV-related cancers.

## Funding

No funding source was provided.

## Declaration of Competing Interest

None.

## References

[1] Anna Szymonowicz K., Chen J. (2020). Biological and clinical aspects of HPV-related cancers. Cancer Biol Med.

[2] CDC HPV Fact Sheet. https://www.cdc.gov/std/hpv/stdfact-hpv.htm.

[3] de Sanjose S., Quint W.G., Alemany L. (2010). Human papillomavirus genotype attribution in invasive cervical cancer: a retrospective cross-sectional worldwide study. Lancet Oncol.

[4] Forman D., de Martel C., Lacey C.J. (2012). Global burden of human papillomavirus and related diseases. Vaccine..

[5] National Cancer Institute. Human Papillomavirus (HPV) Vaccines National Cancer Institute. Published May 25, 2021. https://www.cancer.gov/about-cancer/causes-prevention/risk/infectious-agents/hpv-vaccine-fact-sheet.

[6] Kaiser Family Foundation (KFF) (2021).

[7] Deshmukh A.A., Suk R., Shiels M.S. (2021). Incidence trends and burden of human papillomavirus-associated cancers among women in the United States, 2001-2017. J Natl Cancer Inst.

[8] Backes D.M., Kurman R.J., Pimenta J.M., Smith J.S. (2009). Systematic review of human papillomavirus prevalence in invasive penile cancer. Cancer Causes Control.

[9] Increase the proportion of adolescents who get recommended doses of the HPV vaccine — IID-08. Healthy People 2030. https://health.gov/healthypeople/objectives-and-data/browse-objectives/vaccination/increase-proportion-adolescents-who-get-recommended-doses-hpv-vaccine-iid-08.

[10] Lai J.Y., Tinker Av, Cheung W.Y. (2013). Factors influencing the willingness of US women to vaccinate their daughters against the human papillomavirus to prevent cervical cancer. Med Oncol.

[11] Brewer N.T., Gottlieb S.L., Reiter P.L. (2011). Longitudinal predictors of human papillomavirus vaccine initiation among adolescent girls in a high-risk geographic area. Sex Transm Dis.

[12] Read D.S., Joseph M.A., Polishchuk V., Suss A.L. (2010). Attitudes and perceptions of the HPV vaccine in Caribbean and African-American adolescent girls and their parents. J Pediatr Adolesc Gynecol.

[13] Cummings T., Kasting M.L., Rosenberger J.G., Rosenthal S.L., Zimet G.D., Stupiansky N.W. (2015). Catching up or missing Out? Human papillomavirus vaccine acceptability among 18- to 26-year-old men who have sex with men in a US national sample. Sex Transm Dis.

[14] Licht A.S., Murphy J.M., Hyland A.J., Fix B.V., Hawk L.W., Mahoney M.C. (2010). Is use of the human papillomavirus vaccine among female college students related to human papillomavirus knowledge and risk perception?. Sex Transm Infect.

[15] Pingali C., Yankey D., Elam-Evans L.D. (2021). National, regional, state, and selected local area vaccination coverage among adolescents aged 13–17 years — United States, 2020. MMWR Morb Mortal Wkly Rep.

[16] Boersma P., Black L. (2020).

[17] Auxier B., Anderson M. Social media Use in 2021. https://www.pewresearch.org/internet/2021/04/07/social-media-use-in-2021/.

[18] Lee J., Turner K., Xie Z., Kadhim B., Hong Y.R. (2022). Association between health information–seeking behavior on YouTube and physical activity among U.S. adults: Results From Health Information Trends Survey 2020. AJPM Focus.

[19] Ortiz R.R., Smith A., Coyne-Beasley T. (2019). A systematic literature review to examine the potential for social media to impact HPV vaccine uptake and awareness, knowledge, and attitudes about HPV and HPV vaccination. Hum Vaccin Immunother.

[20] Westat Health Information National Trends Survey 5 (HINTS 5) Cycle 1 - 4 Methodology. Health Information National Trends Survey. Published 2017. https://hints.cancer.gov/docs/methodologyreports/HINTS5_Cycle_1_Methodology_Rpt.pdf.

[21] McBride K.R., Singh S. (2018). Predictors of adults’ knowledge and awareness of HPV, HPV-associated cancers, and the HPV vaccine: implications for health education. Health Educ Behav.

[22] Jo S., Pituch K.A., Howe N. (2022). The relationships between social media and human papillomavirus awareness and knowledge: Cross-sectional study. JMIR Public Health Surveill.

[23] Wheldon C.W., Krakow M., Thompson E.L., Moser R.P. (2019). National trends in human papillomavirus awareness and knowledge of human papillomavirus–related cancers. Am J Prev Med.

[24] Osazuwa-Peters N., Hu A., Rohde R.L. (2018). Sociodemographic predictors of the Human papillomavirus (HPV) and HPV vaccine knowledge and awareness among Americans who use the internet as their primary source of health information. J Consum Health Internet.

[25] Lee H.Y., Luo Y., Daniel C., Wang K., Ikenberg C. (2022). Is HPV vaccine awareness associated with HPV knowledge level? Findings from HINTS data across racial/ethnic groups in the US. Ethn Health.

[26] Le D., Kim H.J., Wen K.Y., Juon H.S. (2022). Disparities in awareness of the HPV vaccine and HPV-associated cancers among racial/ethnic minority populations: 2018 HINTS. Ethn Health.

[27] Bonafide K.E., Vanable P.A. (2015). Male human papillomavirus vaccine acceptance is enhanced by a brief intervention that emphasizes both male-specific vaccine benefits and altruistic motives. Sex Transm Dis.

